# Reproductive status modulates colour preference and multimodal cue integration in host plant location by butterflies

**DOI:** 10.1242/jeb.250414

**Published:** 2025-11-21

**Authors:** K. S. Anaswara, G. S. Balamurali, Hema Somanathan, Ullasa Kodandaramaiah

**Affiliations:** ^1^ IISER-TVM Centre for Research and Education in Ecology and Evolution (ICREEE), School of Biology, Indian Institute of Science Education and Research Thiruvananthapuram, Kerala 695551, India; ^2^ Department of Life Sciences, Atria University, Karnataka 560024, India

**Keywords:** *Catopsilia pomona*, Adaptive plasticity, Oviposition behaviour, Host plants, Phenotypic plasticity

## Abstract

Butterflies utilize visual, odour and tactile cues, individually or in combination, to navigate their surroundings and make decisions. The effectiveness of these cues varies based on distance and context. Colour is a crucial visual cue across multiple behavioural contexts, including when searching for oviposition sites. We investigated the multimodal integration of information and its modulation of colour preference in the common emigrant butterfly, *Catopsilia pomona.* Specifically, we tested their preference towards green colour in different scenarios including during the phase in which they are expected to prioritize finding host plants for oviposition. We offered virgin and mated females and males a choice of four colours including green. We show that mated females prefer green but only in the presence of the odour of their preferred host plant. Virgin females and males, irrespective of mating status, preferred colours other than green. Our results suggest that host plant odour and colour are both salient cues for butterflies, and butterflies use them synergistically to find leaves to oviposit on. The sex-specific preference towards green, and the finding that green is preferred only under some contexts, highlights the adaptive plasticity of colour preferences in butterflies.

## Teaser

**Summary:** Butterfly colour preference is sex specific and varies with behavioural context; butterflies use multimodal integration of sensory cues to locate host plants.

## INTRODUCTION

The selection of host plants by ovipositing females plays a crucial role in the interactions between plants and herbivores and determines the fitness of offspring (Diamond and Kingsolver, 2010; Ehrlich and Raven, 1964; Gibbs et al., 2006; Thompson, 1988). In most insects, larvae are relatively less mobile compared with adults. Therefore, the choice of oviposition substrate by females is critical for the fitness of both parents and offspring. Natural selection favours females that can discern between different hosts based on their suitability for larval development (Carvalho and Vasconcellos-Neto, 2021; Gripenberg et al., 2010; Jaenike, 1978; Videla et al., 2012). For instance, *Polygonia c-album* females prefer to lay eggs on hosts on which their larvae perform best (Gamberale-Stille et al., 2014). Host plant selection involves a series of stages, including searching, orientation, encounter, landing, surface evaluation and acceptance or rejection of the host plant or a specific plant part. During each of these stages, multiple potential cues are available for females, which need to use appropriate cues to choose the most optimal oviposition sites. Inappropriate or inefficient cue usage not only directly affects larval fitness but also imposes a cost in terms of time spent for oviposition.

Females can use visual, chemical and tactile cues to recognize and select appropriate host plants, as well as the best substrate within a chosen plant (Arikawa et al., 2021; Cardé and Willis, 2008; Kelber, 1999; Nagaya et al., 2021; Prokopy and Owens, 1983; Visser, 1982). These cues may exhibit varying degrees of effectiveness depending on the spatial context. During the initial stages of host plant search, plant volatiles may attract the females to the plants (Briscoe et al., 2013). Once on the plant, females employ sensory receptors on their tarsi, antennae, proboscis and ovipositor to evaluate structural and chemical characteristics of the plant surface and determine its suitability for oviposition (Gilbert, 1982; Renwick and Chew, 1994; Thiele et al., 2016), although visual cues may also be important during this stage. Potential visual cues that guide them towards oviposition sites include colour, brightness, shape and polarization (Kelber, 1999; Nagaya et al., 2021).

Females of many phytophagous insects are known to rely on spectral properties such as reflected wavelength to choose oviposition substrates (Ilse, 1937; Kelber, 1999; Kolb and Scherer, 1982; Nagaya et al., 2021; Scherer and Kolb, 1987a; Vaidya, 1969; Weiss and Papaj, 2003; Yoshida et al., 2015). For instance, butterflies of the genus *Pieris* innately prefer green stimuli for oviposition (Ilse, 1937; Kolb and Scherer, 1982; Scherer and Kolb, 1987a). In *Papilio aegues*, oviposition behaviour is guided by a single chromatic mechanism where green receptors positively influence choice behaviour while blue and red receptors negatively influence choice behaviour in the context of oviposition (Kelber, 1999). Furthermore, while searching for a suitable oviposition site, females often depend on innate preferences rather than learned associations (Weiss and Papaj, 2003; but see Braem et al., 2021).

Selection is expected to favour a high degree of plasticity in colour preferences because fitness consequences of preference for a particular colour will depend on the behavioural context (Kolb and Scherer, 1982; Yoshida et al., 2015). Behavioural contexts include foraging, mate choice, oviposition, etc. For example, in butterflies, colour preferences are important in contexts such as mating and foraging (Andersson, 2003; Arikawa, 2017; Arikawa et al., 2021; Kinoshita and Arikawa, 2000; Marini-Filho and Benson, 2010; Ômura and Honda, 2005; Tang et al., 2013). Females and males can identify conspecifics and assess the quality of potential mates based on colour (Blackiston et al., 2011; Li et al., 2017). For instance, females of *Colias eurytheme* use the differences in UV reflectance of males to identify conspecific males (Kemp and Rutowski, 2011; Silberglied, 1984). Unless potential mates are green, a preference for green is maladaptive during courtship. Similarly, the food sources of adult butterflies are flowers, rotting fruit, animal excrement, sap, etc., which are rarely green, and, therefore, butterflies should prefer colours other than green when foraging (Chen et al., 2023). Innate preferences for colours, shapes and odours can aid butterflies in recognizing and perhaps learning of appropriate cues in their environment (Lunau and Maier, 1995; Muñoz-Galicia et al., 2021).

Colour preferences can be sexually dimorphic because males only need to find mates and forage, whereas females need to oviposit in addition to these two tasks. Therefore, unless females are green, males should never prefer green during their adult lifespan, while females should switch to preferring green once they are mated and begin searching for their host plant. For example, *Battus philenor* females show a strong preference for green in the context of oviposition even after training to other colours, whereas males prefer the colours on which they were trained (Weiss and Papaj, 2003). Sexual dimorphism in colour preference has been demonstrated in butterflies such as *Catopsilia pomona*, *Pieris rapae* and *Papilio xuthus* in which females prefer yellow whereas males prefer blue over yellow (Balamurali et al., 2020; Kandori et al., 2009; Kinoshita et al., 1999; Yoshida et al., 2015).

Multiple studies have shown that butterflies can integrate information from different sensory cues and modalities (Balamurali et al., 2019; Yoshida et al., 2015). Tang and colleagues (2013) pointed out four types of signal perception during butterfly foraging and courtship based primarily on vision, primarily on olfaction, equally on vision and olfaction, and on olfaction alone (Tang et al., 2013). For instance, *Papilio xuthus* uses a combination of odour and colour during courtship (Liu et al., 2022). The use of multiple cues provides redundancy and increases the robustness and accuracy of selection (Schäpers et al., 2015). Butterflies exhibit searching behaviour when foraging for flowers, which involves the integration of multiple sensory modalities such as colour vision and olfaction, and butterfly colour preferences can shift based on olfactory contexts (Kinoshita et al., 2017; Yoshida et al., 2015).

Here, we investigated adaptive plasticity in colour preference in a nectar-feeding butterfly, *Catopsilia pomona* (Balamurali et al., 2020). *Catopsilia pomona* has been shown to have true colour vision and can associate a wide range of colours with reward. This species visits flowers for foraging and oviposits on green leaves. We tested the following hypotheses by conducting experiments in which *C. pomona* individuals were allowed to choose stimuli of four different colours in the presence or absence of host plant odour: (1) mated females have a stronger preference for green compared with virgin females; (2) mated females have a stronger preference for green in the presence of the preferred host plant odour than in the absence of the odour; and (3) irrespective of their mating status, males do not show a preference for green.

## MATERIALS AND METHODS

### Butterfly collection and rearing

The experiments were performed on the butterfly *Catopsilia pomona* (Fabricius 1775) (Family Pieridae), a widespread butterfly in tropical Asia, extending into Australia. Eggs and larvae of *C. pomona* from the wild were collected from its host plant, *Cassia fistula* (Nitin et al., 2018), in the Indian Institute of Science Education and Research (IISER) Thiruvananthapuram campus in Vithura, Kerala, India, to establish a laboratory stock population. Larvae of this population feed almost exclusively on *C. fistula* (K.S.A and U.K., personal observation; unpublished data from our lab over 10 years) in the collection locality, Vithura. Therefore, *C. fistula* was considered the preferred host plant for the purposes of this study. Larvae were released in aluminium cages (45×40×40 cm) and provided *ad libitum* with tender leaves of *C. fistula*. After pupation, they were removed from the rearing cages and placed in separate cages for eclosion. Eclosed butterflies were used for experiments.

### Host plant extract

The host plant extract was prepared using tender leaves of *C. fistula* and *Senna alata*, a plant that is not preferred for oviposition by the Vithura *C. pomona* population (K.S.A and U.K., personal observation). Freshly collected leaves were soaked in methanol (1:5 w:v) in glass bottles. The bottles were kept in a shaker incubator (REC-28025-A2, Scigenics Biotech, India) for 7 days (32°C, 180 rpm), following which the leaves were removed, and the extract stored in the refrigerator for later use.

### Visual stimuli

**Fig. 1.**
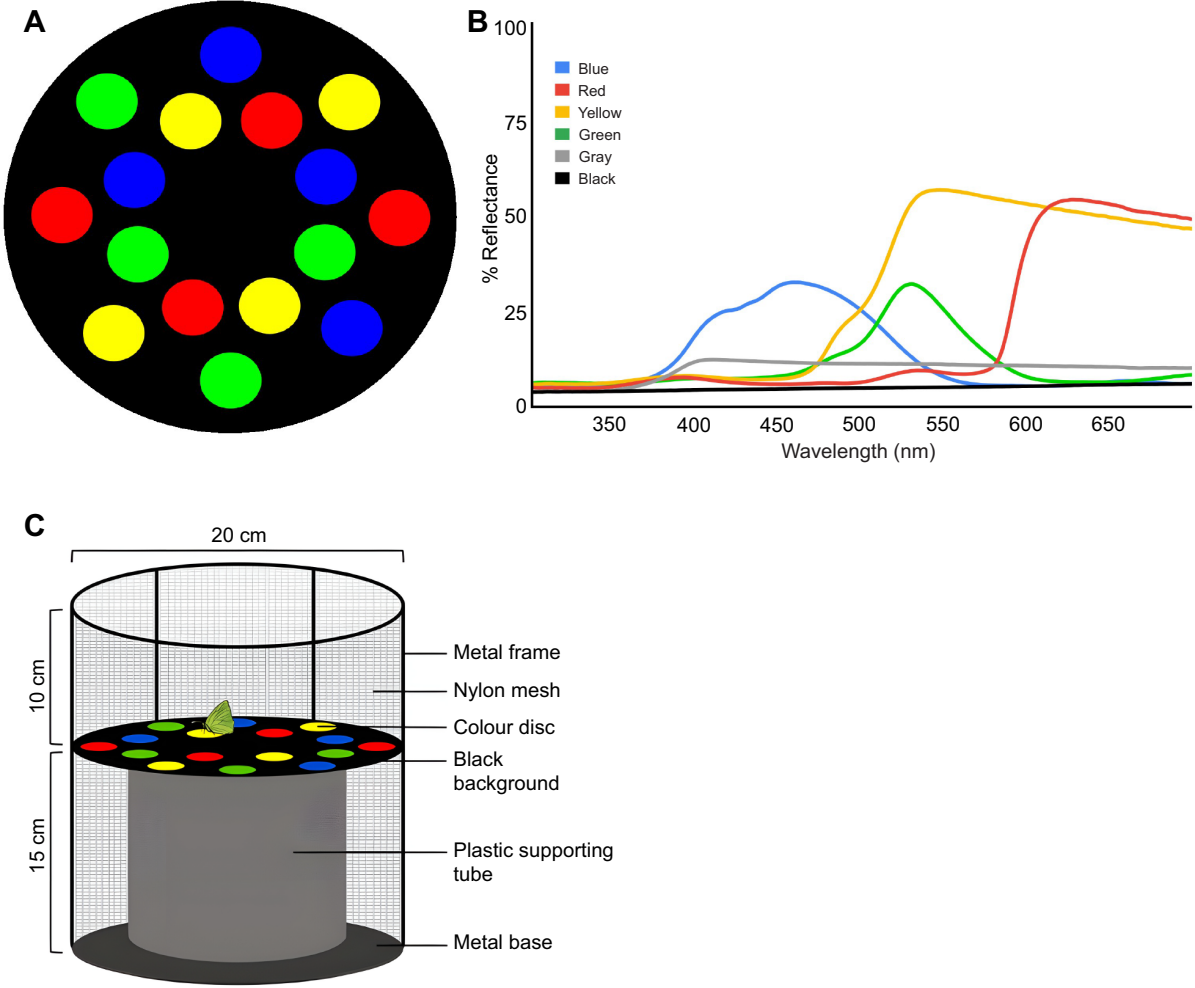
**Experimental setup.** (A) Schematic representation of the four-colour test array presented randomly to test colour preference in *Catopsilia pomona*. (B) Spectral reflectance curves of the four stimuli used. (C) Schematic diagram of the experimental setup.

Coloured vinyl stickers (McSign Coating Technology Inc., Taipei City, Taiwan) stuck on black chart paper cut into circular discs of 4 cm diameter were used to make blue, green, yellow and red stimuli (Fig. 1A). Spectral reflectance of the stimuli (Fig. 1B) was measured with a spectrophotometer (UV-3600i Plus UV-VIS-NIR, Shimadzu, Kyoto, Japan). Four discs of each colour were arranged along two concentric circles in such a way that no two discs of the same colour were placed adjacently (Fig. 1A). Achromatic grey stimuli of the same dimensions were used to check the motivation of butterflies to feed before the trials began.

### Experimental arena

A black nylon mesh-covered cylindrical cage with a metal frame (20 cm in diameter and 25 cm in height) was used as the experimental arena. The top of the cage could be opened to access the arena to let the butterflies in and to change the stimuli. Four of each stimulus were fixed on locally obtained black chart paper (Fig. 1C). The chart paper served as the background and was raised (15 cm) from the base of the cylindrical cage by placing it on a hollow plastic tube, for ease of access.

### Experiments

A total of 3 experiments involving 304 butterflies were conducted outdoors under natural daylight in the IISER Thiruvananthapuram campus. From the stock population, approximately 50% of the newly eclosed individuals were released into separate sex-specific cages, starved for 2 days and used as virgin individuals for the experiment. The rest of the eclosed individuals were fed with 20% sucrose solution and were released together into a different cage for mating. They were starved for 2 days after mating before conducting the experiment.

All individuals were allowed to feed for 5 s on the achromatic grey stimulus with 20% sucrose solution before experiments, to test their motivation. Fed individuals were released into test cages. Each individual was tested only once. The position of the colour stimuli was changed randomly in each trial to prevent any positional bias. In addition, stimuli were wiped with 70% ethanol between trials to avoid olfactory cues from the previous test. A butterfly was considered to have responded to a particular stimulus if it landed on the stimulus and extended its proboscis. The first visit and visits made in the subsequent 3 min were recorded for each butterfly tested.

The experiments were done with virgin and mated males and females (1) in the absence of added odour, (2) in the presence of preferred host plant odour (*C. fistula*) and (3) in the presence of non-preferred host plant odour (*S. alata*) as detailed below.

#### Colour preference of virgin and mated males and females in the absence of odour

After 2 days of starvation, virgin females (*n*=28), virgin males (*n*=34), mated females (*n*=22) and mated males (*n*=28) were individually released into the experimental arena containing four stimuli each of blue, green, yellow and red. The visits of each individual were noted.

#### Colour preference of virgin and mated males and females in the presence of preferred host plant odour

Starved (for 2 days) virgin females (*n*=31), virgin males (*n*=25), mated females (*n*=26) and mated males (*n*=28) were released into the experimental arena. The colour stimuli were coated with 1 ml extract of *C. fistula* using a cotton ball. The visits were noted as described above.

#### Colour preference of virgin and mated males and females in the presence of non-preferred host plant odour

Virgin females (*n*=20), virgin males (*n*=26), mated females (*n*=19) and mated males (*n*=17) starved for 2 days were released into the experimental arena containing colour stimuli coated with 1 ml extract of *S. alata*, and visits were noted as mentioned above.

### Statistical analysis

All statistical analyses and visualization were performed in R v.4.1.2 ‘Bird Hippie’ (http://www.R-project.org/) via R Studio v.2023.6.1.524 ‘Mountain Hydrangea’ (http://www.posit.co/). There was variation in the number of total visits made by individual butterflies within experiments. For all experiments, the first visits and the total visits of individual butterflies were analysed separately. Chi-square tests were performed to determine whether the number of first visits differed significantly across colours. For each female, the proportion of total visits to the four colours was calculated, and the proportions were analysed to test for differences in total visits among colours. Kruskal–Wallis tests followed by *post hoc* pair-wise comparisons using Dunn's test with Bonferroni correction were used to test for differences in total visits between pairs of colours.

ChatGPT (OpenAI 2024) was used to generate codes to analyse the data in R. The authors subsequently reviewed and edited the content as necessary and take full responsibility for the publication's final content.

## RESULTS

### Colour preference of virgin and mated males and females in the absence of added odour

**Fig. 2.**
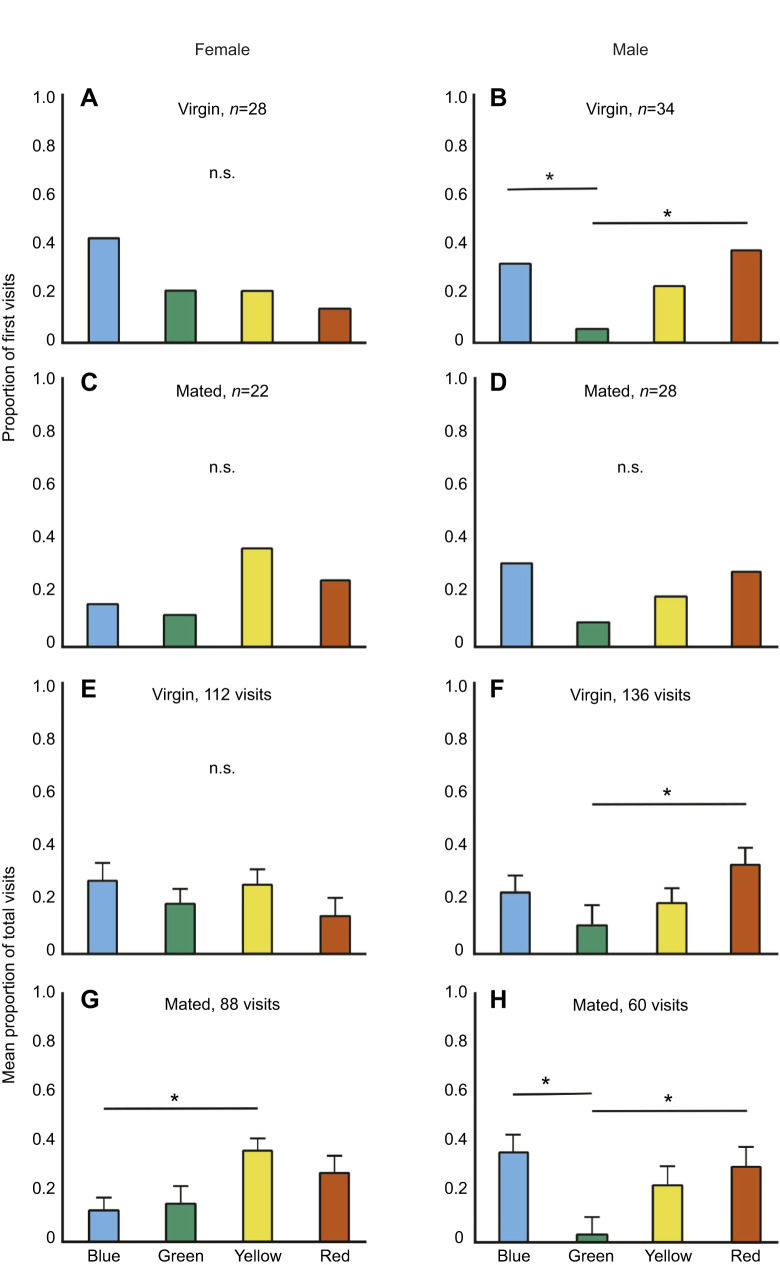
**Proportion of first visits and mean proportion of total visits in the absence of added odour.** Proportion of first visits made by (A) virgin females, (B) virgin males, (C) mated females and (D) mated males. Mean (±s.e.m.) proportion of total visits made by (E) virgin females, (F) virgin males, (G) mated females and (H) mated males. For the first visits, *n* indicates the number of individuals tested. n.s. indicates no significant overall difference among colours in a comparison (Chi-square tests for first visits; Kruskal–Wallis for total visits). Pairwise comparisons between colours were done when there was an overall significant difference, and in these cases, an asterisk indicates a significant pairwise difference (Chi-square test for first visits; Dunn's *post hoc* test for total visits).

The number of first visits made by virgin females did not differ from random (i.e. 25%) across the four colours (*n*=28; χ^2^=5.14, d.f.=3, *P*=0.1616; Fig. 2A) whereas first visits by virgin males were different from random (*n*=34; χ^2^=8.11, d.f.=3, *P*=0.0436; Fig. 2B). Pairwise comparisons indicated that first visits by virgin males were primarily to red (38%) or blue (32%) (see Table S2). The first visits were random in mated females (*n*=22; χ^2^=3.81, d.f.=3, *P*=0.2818; Fig. 2C) and mated males (*n*=28; χ^2^=4.28, d.f.=3, *P*=0.2322; Fig. 2D).

The total number of visits by virgin females did not differ across the four colours (112 visits; *H*=5.83, d.f.=3, *P*=0.12; Fig. 2E), whereas the total number of visits differed across the colours in virgin males (136 visits; *H*=10.68, d.f.=3, *P*=0.0136; Fig. 2F), mated females (88 visits; *H*=11.25, d.f.=3, *P*=0.0104; Fig. 2G) and mated males (60 visits; *H*=13.26, d.f.=3, *P*=0.0041; Fig. 2H). Pairwise comparisons of total visits showed that virgin males primarily preferred red, mated females preferred yellow, and mated males preferred blue and red (see Tables S1 and S2).

### Colour preference of virgin and mated males and females in the presence of preferred host plant odour

**Fig. 3.**
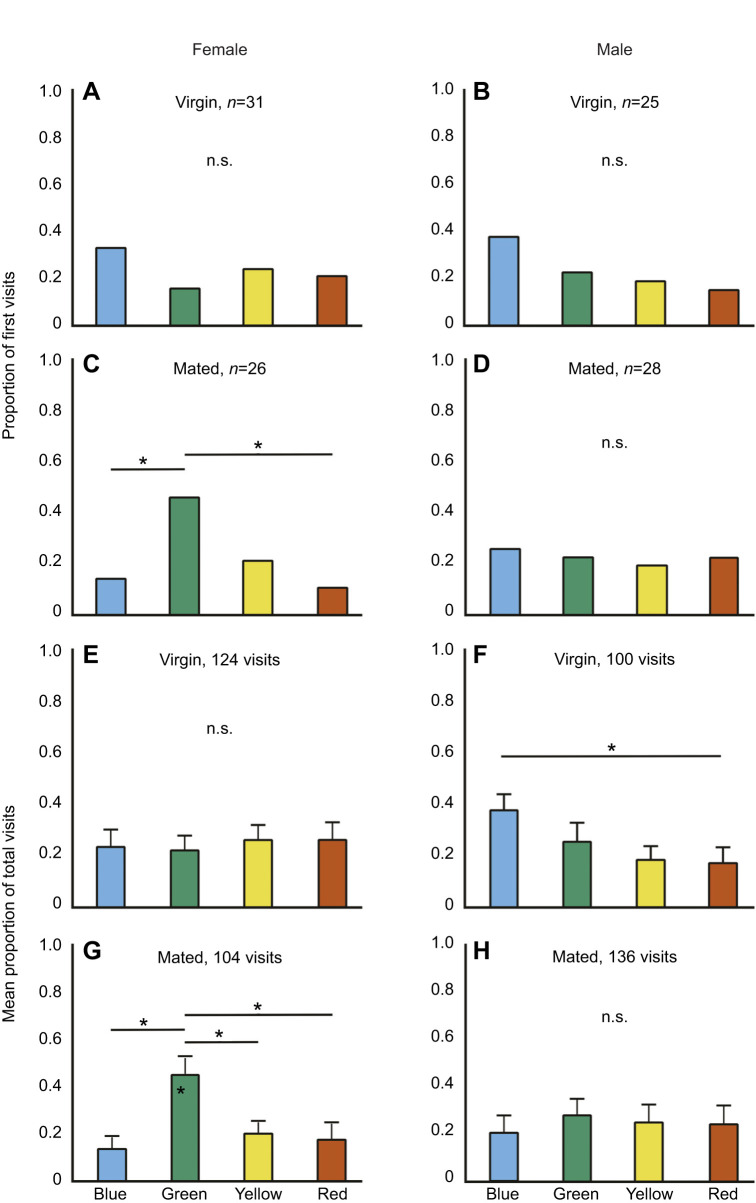
**Proportion of first visits and mean proportion of total visits in the presence of preferred host plant odour.** Proportion of first visits made by (A) virgin females, (B) virgin males, (C) mated females and (D) mated males. Mean proportion of total visits made by (E) virgin females, (F) virgin males, (G) mated females and (H) mated males. For the first visits, *n* indicates the number of individuals tested. n.s. indicates no significant overall difference among colours in a comparison (Chi-square tests for first visits; Kruskal–Wallis for total visits). Pairwise comparisons between colours were done when there was an overall significant difference, and in these cases, an asterisk indicates a significant pairwise difference (Chi-square test for first visits; Dunn's *post hoc* test for total visits).

First visits across the four colours by virgin females (*n*=31; χ^2^=2.41, d.f.=3, *P*=0.49; Fig. 3A) and males (*n*=25; χ^2^=3.32, d.f.=3, *P*=0.3449; Fig. 3B) were random. The number of first visits made by mated females was different from random (*n*=26; χ^2^=9.38, d.f.=3, *P*=0.0245; Fig. 3C) while the first visits by mated males were random (*n*=28; χ^2^=0.28, d.f.=3, *P*=0.9627; Fig. 3D). Pairwise comparison of mated females indicated that they preferred green over blue and red (see Table S3).

The number of total visits did not differ across the four colours in virgin females (124 visits; *H*=1.19, d.f.=3, *P*=0.754; Fig. 3E), whereas virgin males (100 visits; *H*=9.945, d.f.=3, *P*=0.019; Fig. 3F) primarily chose blue. Total visits across the four colours differed for mated females (104 visits; *H*=27.42, d.f.=3, *P*<0.00001; Fig. 3G) but not for mated males (136 visits; *H*=10.68, d.f.=3, *P*=0.674; Fig. 3H). Pairwise comparisons indicated that virgin males primarily preferred blue and mated females preferred green (see Table S4).

### Colour preference of virgin and mated males and females in the presence of non-preferred host plant odour

**Fig. 4.**
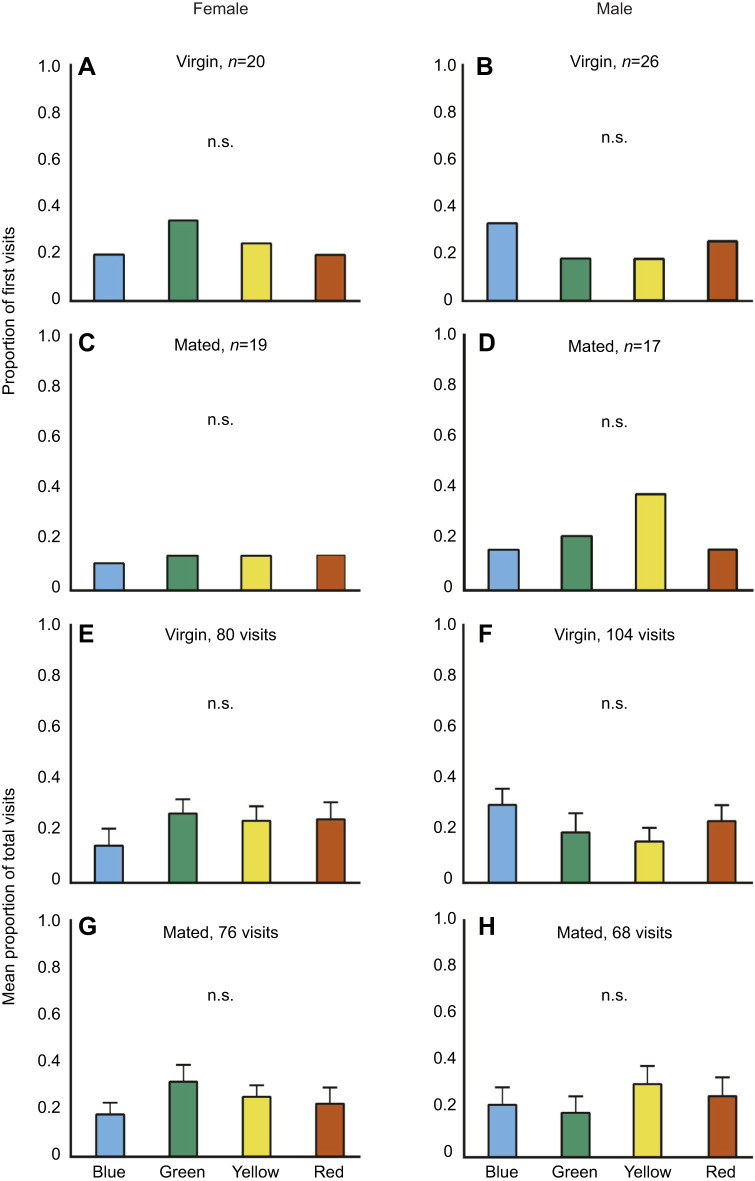
**Proportion of first visits and mean proportion of total visits in the presence of non-preferred host plant odour.** Proportion of first visits made by (A) virgin females, (B) virgin males, (C) mated females and (D) mated males. Mean proportion of total visits made by (E) virgin females, (F) virgin males, (G) mated females and (H) mated males. For the first visits, *n* indicates the number of individuals tested. n.s. indicates no significant overall difference among colours in a comparison (Chi-square tests for first visits; Kruskal–Wallis for total visits). Pairwise comparisons between colours were done when there was an overall significant difference, and in these cases, an asterisk indicates a significant pairwise difference (Chi-square test for first visits; Dunn's *post hoc* test for total visits).

The number of first visits by virgin females (*n*=20; χ^2^=1.2, d.f.=3, *P*=0.753; Fig. 4A), virgin males (*n*=26; χ^2^=1.69, d.f.=3, *P*=0.6386; Fig. 4B), mated females (*n*=19; χ^2^=0.15, d.f.=3, *P*=0.9841; Fig. 4C) and mated males (*n*=17; χ^2^=2.52, d.f.=3, *P*=0.47; Fig. 4D) did not differ from random (see Tables S5 and S6).

Total visits by virgin females (80 visits; *H*=3.5786, d.f.=3, *P*=0.311; Fig. 4E), virgin males (104 visits; *H*=3.22, d.f.=3, *P*=0.358; Fig. 4F), mated females (76 visits; *H*=1.30, d.f.=3, *P*=0.727; Fig. 4G) and mated males (68 visits; *H*=1.1044, d.f.=3, *P*=0.776; Fig. 4H) did not differ across the four colours (see Tables S5 and S6).

## DISCUSSION

Our results support the idea that colour preference in butterflies is plastic rather than genetically hardwired. Behavioural contexts such as foraging, seeking mates or finding a suitable oviposition site modulate the expression of colour preferences in butterflies. This flexibility allows the butterflies to optimize their behaviour based on varying internal and external conditions, thus maximizing their fitness. We show that *C. pomona* has plasticity in preference for green colour. Green is an important visual cue often associated with host plants, making it highly relevant in the context of oviposition by females (Balamurali et al., 2020; Hirota and Kato, 2004; Kelber, 1999; Nagaya et al., 2021; Scherer and Kolb, 1987a,b). Green flowers are rare in nature (Dyer et al., 2021; Wang et al., 2022; Weevers, 1952), and green is expected to be primarily associated with oviposition (Scherer and Kolb, 1987a). However, a recent study (Del Valle et al., 2025) suggests that many green flowers possess a yellowish tinge, which can enhance their chromatic contrast to the green foliage background. Notwithstanding this, a generally held view is that a preference for green is expected to be maladaptive in all contexts except oviposition (Kelber, 1999; Prokopy and Owens, 1983). Female butterflies do not associate green with reward even after prolonged training (Balamurali et al., 2020; Weiss and Papaj, 2003). Our results show that mated *C. pomona* females exhibited a strong preference for green, while virgin females chose colours such as blue and red, which are associated with nectar-rewarding flowers (Fig. 3). In *C. pomona*, this preference towards green may be particularly adaptive as its larvae feed exclusively on the tender leaves of host plants and not on mature leaves (K.S.A and U.K., personal observation). Females may differentiate between tender and mature leaves based solely on subtle variations in the spectral reflectance. For example *Helicoverpa armigera* (Liu et al., 2022) rely more on brightness than on colour for oviposition selection (also see Chen et al., 2021b). Exploring whether females can discern these nuances in green could provide valuable insights into how visual cues contribute to host plant selection in butterflies.

Before mating, females are expected to prioritize foraging and mate search. In our study, virgin females chose colours such as yellow, blue and red, which are associated with nectar-rewarding flowers (Fig. 3). The preference towards yellow in females in *C. pomona* may also be a sensory bias to recognize conspecifics because wings of both sexes of this species reflect broadly in the yellow region of the visible spectrum (Balamurali et al., 2020; also see Blackiston et al., 2011). However, males preferred red and blue over yellow (Fig. 2), suggesting that the yellow preference is unlikely to be explained by mate selection alone. Intriguingly, the preference for red and blue was not evident in males in the experiments where plant odour was added (Fig. 3), suggesting that plant odour interacts with other sensory cues to influence colour preference. The sexual dimorphism in colour preference, wherein females preferred yellow while males preferred red and blue (Fig. 2), warrants further investigation. Interestingly, in many butterfly species there is sexual dimorphism in the peak sensitivities of photoreceptors and in the screening pigments in the ommatidia which indirectly shift peak photoreceptor sensitivity. For example, in *Pieris rapae*, the peak sensitivity of the photoreceptor sensitive to violet differs between males and females (Arikawa et al., 2005). This is hypothesized to help males identify mates. Furthermore, in *Colias erate*, screening pigments in the ventral eye region shift the peak sensitivity of the red pigment in the photoreceptors of the males to 660 nm from that of 620 nm in females (Ogawa et al., 2013). As the ventral region of the eye plays a crucial role in visually guided sexual behaviours, such as mate choice and oviposition, males and females might use the input from the red receptor differently.

Our study shows how mating status can influence colour preference. After mating, females should prioritize finding a suitable oviposition site. The shift in colour preference towards green post-mating (Fig. 3) highlights the role of behavioural context (Nagaya et al., 2021; Weiss and Papaj, 2003). Balamurali and colleagues (2020) showed that virgin females show a strong aversion towards the colour green in the context of foraging. Interestingly, in experiments where naive butterflies were trained to green, i.e. when green was associated with a nectar reward, males preferred green more strongly than did females, suggesting that it is difficult for females to learn to associate green with behavioural contexts other than oviposition.

In our study, there was no preference for green when the odour of the host plant was absent (Fig. 2), underscoring the importance of multimodal integration of sensory cues in decision making. Butterflies are known to rely on multiple sensory cues, including visual, chemical and tactile, in the context of oviposition (Schäpers et al., 2015). They can use these cues either individually or in combination (Balamurali et al., 2019; Cepero et al., 2015; Costanzo and Monteiro, 2007; Dell'Aglio et al., 2016; Weiss and Papaj, 2003; Yoshida et al., 2015). For instance, mated *Aglais urticae* and *Polygonia c-album* females navigate towards their host plant using odour cues (Schäpers et al., 2015). Mated *Battus philenor* females use a combination of visual and chemical cues to find oviposition sites (Weiss and Papaj, 2003). In contrast, *Papilio maackii* females rely primarily on olfactory cues when selecting flowers while foraging (Chen et al., 2021a). Integration of these signals may depend on the distance between the butterfly and the target. For instance, from a distance, females may rely on visual cues such as substrate colour, the height of the­ substrate from the ground or substrate shape to navigate towards potential targets. Once in closer proximity to the substrate, they may use chemical or tactile cues to choose the optimal site for oviposition. Alternatively, they may detect chemical cues from a distance, which guides them toward a specific area, and then use colour to pinpoint the target. It is also plausible that butterflies use a combination of colour and odour at all stages when locating their host plant. In our study, we found that mated females preferred green in the presence of preferred host plant odour (Fig. 3), but not when there was no added odour (Fig. 2) or when the odour of a non-preferred host plant was added (Fig. 4). In our experiments, butterflies were released in close proximity to the coloured discs on which they landed. Therefore, our results suggest that the presence of host plant odour is important even in close proximity. Females may ultimately choose oviposition substrates based on a combination of colour and odour but will not inspect a green substrate unless the correct host plant odour is also present.

Because males do not oviposit, and both sexes of this species have yellow wings, they are unlikely to prefer green under any behavioural context. As predicted, *C. pomona* males never preferred green, irrespective of their mating status (Figs 2, 3 and 4). Our study is the first to show sex-specific differences in preference for green. This difference in behaviour indicates that males and females have evolved distinct strategies based on their reproductive roles (Weiss, 1997). Even though males do not oviposit, they may locate their mating partners by searching near host plants (Tiple et al., 2010). For example, males of the butterfly *Phengaris teleius* exhibit longer and less regular flights to find females near their larval host plants (Popović et al., 2022). The absence of green preference in *C. pomona* males suggests that males of this species do not rely on host plant cues to locate females. Intriguingly, odour modulated the colour preferences in males, but differently from that in females. When odour was added – either of the host plant or of a non-host plant – there was no preference towards any colour (Figs 3 and 4), suggesting sex-specific effects of sensory integration. This loss of responsiveness to colour in the presence of odour suggests that colour and odour cues act antagonistically during decision making in males. *In Papilio xuthus*, both males and females prefer blue in the absence of odour cues. However, in the presence of floral scents such as neroli and lilio, female preference for red increases, while male preference does not change. When the floral scents are replaced with the scent of their host plant, female preference for green increases, while male preference remains unaffected (Yoshida et al., 2015). This suggests that there is sexual dimorphism in sensory integration (Kinoshita et al., 2017).

### Summary and conclusions

Our study suggests that colour preference in butterflies is sexually dimorphic and influenced by the context in which the preference is shown. Virgin females preferred flower-associated colours such as yellow and blue, presumably because these colours are important in a foraging context. A preference for yellow may also be important to locate and identify mates in this species. Mated females exhibited a strong preference for green, but this preference for green was seen only when host plant odour was also present, indicating that butterflies integrate both colour and odour cues when choosing oviposition substrates, at least when they are in close proximity to potential substrates. Our findings indicate that host plant selection in this species is influenced by the integration of multiple sensory modalities. Physiological states alone are insufficient to trigger behavioural changes unless they are accompanied by the integration of diverse environmental cues. Males, in contrast, exhibit no preference towards green, and this is expected to be adaptive because males do not oviposit. These sex-specific and behavioural context-driven modulations demonstrate the plasticity of colour preference in butterflies as well as the interplay of visual and chemical cues in butterfly decision making.

## Supplementary Material

10.1242/jexbio.250414_sup1 Supplementary information
