# Supplementary material for: Reproductive status modulates colour preference and multimodal cue integration in host plant location by butterflies
Source: J Exp Biol. 2025 Nov 21;228(22):jeb250414. doi: 10.1242/jeb.250414 (PMC12669839; doi:10.1242/jeb.250414)
Supplement: Supplementary information [file jexbio-228-250414-s1.pdf]

Table S1. Pair-wise comparisons for A) virgin females (n=28), and B) mated females (n=22) in the absence of added odour. Chi-square tests were done to compare first visits and Dunn's tests with Bonferroni correction to compare total visits. Significant results are in bold font.

Females				
Comparisons	A) Virgin		B) Mated	
	First visit Chi-square tests	Total visits Dunn's tests	First visit Chi-square tests	Total visits Dunn's tests
Blue *vs* Green	$\chi 2$ = 2, df = 1, p = 0.157	df = 3, *p*=0.621	$\chi 2$ = 0.142, df = 1, p = 0.705	df = 3, *p*=1
Blue *vs* Yellow	$\chi 2$ = 2, df = 1, p = 0.157	df = 3, *p*=1	$\chi 2$ = 1.923, df = 1, p = 0.165	**df = 3, *p*=0.0184**
Blue *vs* Red	$\chi 2$ = 4, df = 1, p = 0.045	df = 3, *p*=0.317	$\chi 2$ = 0.4, df = 1, p = 0.527	df = 3, *p*=0.368
Green *vs* Yellow	$\chi 2$ = 0, df = 1, p = 1	df = 3, *p*=0.893	$\chi 2$ = 3, df = 1, p = 0.832	df = 3, *p*=0.0585
Green *vs Red*	$\chi 2$ = 0.4, df = 1, p = 0.527	df = 3, *p*=1	$\chi 2$ = 1, df = 1, p = 0.317	df = 3, *p*=0.810
Yellow *vs* Red	$\chi 2$ = 0.4, df = 1, p = 0.527	df = 3, *p*=0.479	$\chi 2$ = 0.6, df = 1, p = 0.438	df = 3, *p*=1

Journal of Experimental Biology • Supplementary information

Table S2. Pair-wise comparisons for A) virgin males (n=34), and B) mated males (n=28) in the absence of added odour. Chi-square tests were done to compare first visits and Dunn's tests with Bonferroni correction to compare total visits. Significant results are in bold font.

Males				
Comparisons	A) Virgin		B) Mated	
	First visit Chi-square tests	Total visits Dunn's tests	First visit Chi-square tests	Total visits Dunn's tests
Blue *vs* Green	**χ2 = 6.230, df = 1, p = 0.012**	df = 3, *p*=0.445	χ2 = 3.769, df = 1, p = 0.052	**df = 3, *p*=0.00579**
Blue *vs* Yellow	χ2 = 0.473, df = 1, p = 0.491	df = 3, *p*=1	χ2 = 1, df = 1, p = 0.317	df = 3, *p*=0.846
Blue *vs* Red	χ2 = 0.166, df = 1, p = 0.683	df = 3, *p*=0.845	χ2 = 0.052, df = 1, p = 0.818	df = 3, *p*=1
Green *vs* Yellow	χ2 = 3.60, df = 1, p = 0.057	df = 3, *p*=0.748	χ2 = 1, df = 1, p = 0.317	df = 3, *p*=0.405
Green *vs Red*	**χ2 = 8.06, df = 1, p = 0.004**	**df = 3, *p*=0.00672**	χ2 = 3, df = 1, p = 0.083	**df = 3, *p*=0.0187**
Yellow *vs* Red	χ2 = 1.190, df = 1, p = 0.275	df = 3, *p*=0.509	χ2 = 0.6, df = 1, p = 0.438	df = 3, *p*=1

Journal of Experimental Biology • Supplementary information

Table S3. Pair-wise comparisons for A) virgin females (n=31), and B) mated females (n=26) in the presence of preferred host plant odour. Chi-square tests were done to compare first visits and Dunn's tests with Bonferroni correction to compare total visits. Significant results are in bold font.

Females				
Comparisons	A) Virgin		B) Mated	
	First visit Chi-square tests	Total visits Dunn's tests	First visit Chi-square tests	Total visits Dunn's tests
Blue *vs* Green	$\chi 2$ = 2.250, df = 1, p = 0.133	df = 3, *p*=0.6	**$\chi 2$ = 4.764, df = 1, p = 0.029**	**df = 3, p=0.0000357**
Blue *vs* Yellow	$\chi 2$ = 0.473, df = 1, p = 0.491	df = 3, p=0.621	$\chi 2$ = 0.400, df = 1, p = 0.527	df = 3, p=1
Blue *vs* Red	$\chi 2$ = 0.888, df = 1, p = 0.345	df = 3, p=0.738	$\chi 2$ = 0.142, df = 1, p = 0.705	df = 3, p=1
Green *vs* Yellow	$\chi 2$ = 0.692, df = 1, p = 0.405	df = 3, p=0.308	$\chi 2$ = 2.578, df = 1, p = 0.108	**df = 3, p=0.0000519**
Green *vs Red*	$\chi 2$ = 0.333, df = 1, p = 0.563	df = 3, p=0.849	**$\chi 2$ = 6.250, df = 1, p = 0.012**	**df = 3, p=0.0000108**
Yellow *vs* Red	$\chi 2$ = 0.066, df = 1, p = 0.796	df = 3, p=0.407	$\chi 2$ = 1, df = 1, p = 0.317	df = 3, p=1

Table S4. Pair-wise comparisons for A) virgin males (n=25), and B) mated males (n=28) in the presence of preferred host plant odour. Chi-square tests were done to compare first visits and Dunn's tests with Bonferroni correction to compare total visits. Significant results are in bold font.

Males				
Comparisons	A) Virgin		B) Mated	
	First visit Chi-square tests	Total visits Dunn's tests	First visit Chi-square tests	Total visits Dunn's tests
Blue *vs* Green	$\chi 2$ = 1, df = 1, p = 0.317	df = 3, p=0.998	$\chi 2$ = 0.666, df = 1, p = 0.796	df = 3, p=1
Blue *vs* Yellow	$\chi 2$ = 1.666, df = 1, p = 0.196	df = 3, p=0.183	$\chi 2$ = 0.285, df = 1, p = 0.592	df = 3, p=1
Blue *vs* Red	$\chi 2$ = 2.571, df = 1, p = 0.108	**df = 3, p=0.0145**	$\chi 2$ = 0.066, df = 1, p = 0.796	df = 3, p=1
Green *vs* Yellow	$\chi 2$ = 0.090, df = 1, p = 0.763	df = 3, p=1	$\chi 2$ = 0.076, df = 1, p = 0.781	df = 3, p=1
Green *vs Red*	$\chi 2$ = 0.400, df = 1, p = 0.527	df = 3, p=0.593	$\chi 2$ = 0, df = 1, p = 1	df = 3, p=1
Yellow *vs* Red	$\chi 2$ = 0.111, df = 1, p = 0.738	df = 3, p=1	$\chi 2$ = 0.076, df = 1, p = 0.781	df = 3, p=1

Journal of Experimental Biology • Supplementary information

Table S5. Pair-wise comparisons for A) virgin females (n=20), and B) mated females (n=19) in the presence of non-preferred host plant odour. Chi-square tests were done to compare first visits and Dunn's tests with Bonferroni correction to compare total visits. Significant results are in bold font.

Females				
Comparisons	A) Virgin		B) Mated	
	First visit Chi-square tests	Total visits Dunn's tests	First visit Chi-square tests	Total visits Dunn's tests
Blue *vs* Green	$\chi 2$ = 0.818, df = 1, p = 0.365	df = 3, p=0.450	$\chi 2$ = 0, df = 1, p = 1	df = 3, p=1
Blue *vs* Yellow	$\chi 2$ = 0.111, df = 1, p = 0.738	df = 3, p=1	$\chi 2$ = 0, df = 1, p = 1	df = 3, p=1
Blue *vs* Red	$\chi 2$ = 0, df = 1, p = 1	df = 3, p=0.894	$\chi 2$ = 0, df = 1, p = 1	df = 3, p=1
Green *vs* Yellow	$\chi 2$ = 0.333, df = 1, p = 0.563	df = 3, p=1	$\chi 2$ = 0, df = 1, p = 1	df = 3, p=1
Green *vs Red*	$\chi 2$ = 0.818, df = 1, p = 0.365	df = 3, p=1	$\chi 2$ = 0, df = 1, p = 1	df = 3, p=1
Yellow *vs* Red	$\chi 2$ = 0.111, df = 1, p = 0.738	df = 3, p=1	$\chi 2$ = 0, df = 1, p = 1	df = 3, p=1

Journal of Experimental Biology • Supplementary information

Table S6. Pair-wise comparisons for A) virgin males (n=26), and B) mated males (n=17) the presence of non-preferred host plant odour. Chi-square tests were done to compare first visits and Dunn's tests with Bonferroni correction to compare total visits. Significant results are in bold font.

Males				
Comparisons	A) Virgin		B) Mated	
	First visit Chi-square tests	Total visits Dunn's tests	First visit Chi-square tests	Total visits Dunn's tests
Blue *vs* Green	$\chi 2$ = 1.142, df = 1, p = 0.285	df = 3, p=1	$\chi 2$ = 0.142, df = 1, p = 0.705	df = 3, p=1
Blue *vs* Yellow	$\chi 2$ = 1.142, df = 1, p = 0.285	df = 3, p=.459	$\chi 2$ = 1.600, df = 1, p = 0.205	df = 3, p=1
Blue *vs* Red	$\chi 2$ = 0.250, df = 1, p = 0.617	df = 3, p=1	$\chi 2$ = 0, df = 1, p = 1	df = 3, p=1
Green *vs* Yellow	$\chi 2$ = 0, df = 1, p = 1	df = 3, p=1	$\chi 2$ = 0.818, df = 1, p = 0.365	df = 3, p=1
Green *vs Red*	$\chi 2$ = 0.333, df = 1, p = 0.563	df = 3, p=1	$\chi 2$ = 0.142, df = 1, p = 0.705	df = 3, p=1
Yellow *vs* Red	$\chi 2$ = 0.333, df = 1, p = 0.563	df = 3, p=1	$\chi 2$ = 1.600, df = 1, p = 0.205	df = 3, p=1

Journal of Experimental Biology • Supplementary information
